# Optimality as a framework for understanding developmental robustness

**DOI:** 10.1016/j.tcb.2025.08.009

**Published:** 2026-03

**Authors:** Prachiti Moghe, Edouard Hannezo, Takashi Hiiragi

**Affiliations:** 1 Hubrecht Institute, Royal Netherlands Academy of Arts and Sciences (KNAW), Utrecht, The Netherlands; 2 Developmental Biology Unit, European Molecular Biology Laboratory, Heidelberg, Germany; 3 Institute of Science and Technology Austria, Klosterneuburg, Austria; 4 Institute for the Advanced Study of Human Biology (WPI-ASHBi), Kyoto University, Kyoto, Japan; 5 Department of Developmental Biology, Graduate School of Medicine, Kyoto University, Kyoto 606-8501, Japan

**Keywords:** patterning precision, embryogenesis, morphogenesis, quantitative biology, mechanochemical feedback, tuning of control parameters

## Abstract

Embryo growth, morphogenesis, and patterning are complex processes that coordinate between cellular dynamics, fate specification, and multiscale physical forces. Understanding how robustness in embryo development is achieved despite inherent heterogeneities in gene expression, cell properties, and tissue growth is a fundamental question. Although various feedback between gene expression, signaling, and cell and tissue mechanics have been uncovered to confer robustness on developmental systems, measuring variability and robustness from a quantitative perspective often remains challenging. Furthermore, cell fate plasticity, a key mechanism that can confer robustness, is lacking in many developing tissues. This review highlights how recent technological and conceptual advances in quantitative approaches to biology help to overcome these bottlenecks, with a particular focus on how mechanochemical feedback, or alternatively, selectively tuned control parameters, ensure developmental robustness.

## Highlights

Understanding the robustness of complex traits *in vivo* is challenging due to inherent stochastic behaviour and difficulties in capturing variabilities across scales.
Advances in quantitative methods have improved our ability to analyze biological variability and gain deeper insights into developmental processes.
Feedback mechanisms operating at the molecular, cellular, and tissue levels can ensure robustness in embryonic patterning.
In developing tissues lacking feedback (regulatory mechanisms that control responses) and plasticity (capacity for alternative developmental outcomes), coupling of key parameters through evolutionary adaptation can confer developmental robustness.

## Robustness in embryo patterning and morphogenesis

Robustness is a property that allows the generation and maintenance of a particular outcome or function despite internal noise and external perturbations. The development of multicellular organisms is a striking example of such robustness. Embryo patterning and morphogenesis occur while embryos increase in size and complexity by many orders of magnitude, buffering various types of noise and stochastic variations [1,2]. Despite these challenges, organisms consistently self-organize into reproducible shapes and patterns with specific functions, while steadily processing energy, maintaining homeostasis, adapting to changing environments, and reproducing for continued survival. The success of embryonic development therefore requires an intricate coordination of the mechanisms that pattern an embryo with a variety of cellular fates, and those that sculpt tissues into their complex 3D shapes.

Understanding robustness in development has been challenging due to a number of experimental and conceptual limitations. First, during development, noise can arise from many different types of intrinsic or extrinsic sources, and their quantification remains difficult. Gene expression is intrinsically noisy due to small-number fluctuations at the molecular level, imposing fundamental bounds on how reproducible cellular processes can be [3., 4., 5.]. External fluctuations including metabolic supply, availability of nutrients, and temperature, also need to be buffered – at least within a certain conducive range. Moreover, natural genetic variation, including mutations and polymorphisms, may alter gene dosage. Patterning and morphogenesis must be coordinated with embryo growth to ensure that cell-type proportions and tissue shapes can withstand natural variations and fluctuations in growth rates. Second, a prominent challenge is to define the robustness of complex traits *in vivo*, such as a cell fate pattern or a high-dimensional morphological structure, and many findings rely on fixed biological samples, lacking insights into how variability evolves in time. Third, investigating robustness in development necessitates not only the quantitative analysis of a large number of samples, but also control over different system parameters to distinguish between intrinsic and extrinsic, as well as technical, sources of variability.

In developmental biology, some of these challenges are beginning to be overcome. A possible solution to define the robustness of complex qualitative traits involves a statistical approach, in which a large number of samples is analyzed and the observed phenotypes are quantified in the absence and presence of controlled perturbations. This approach goes beyond the classification of phenotypes as merely ‘normal’ or ‘deviant’. Recent improvements in imaging and image analysis have also emerged as powerful tools to generate comprehensive datasets of developmental processes and to quantify biological variability at different scales [6., 7., 8.].

Quantitative approaches towards developmental processes have provided deeper insights, but have also revealed gaps in our understanding; for example, in biological contexts that lack feedback mechanisms. This review discusses recent insights into the mechanisms that ensure robustness in patterning and morphogenesis, first those involving feedback interactions, and then introduces emerging evidence of those without feedback, highlighting how these systems nevertheless achieve robustness in biological function.

## Feedback interactions in cell fate specification and patterning

Advances in quantitative study have revealed that, despite inherent variabilities in biological systems, feedback mechanisms operating at the molecular, cellular, and tissue levels can ensure robustness and precision in embryonic patterning. These key properties of living systems, while related, may vary independently. Robustness is the maintenance of consistent outcomes despite fluctuations, genetic variations, or other perturbations, whereas precision is the accuracy and fine-scale resolution with which spatial patterns, temporal sequences, and cell fates are established under certain conditions.

### Mechanical coupling coordinates morphogenesis

**Figure 1:**
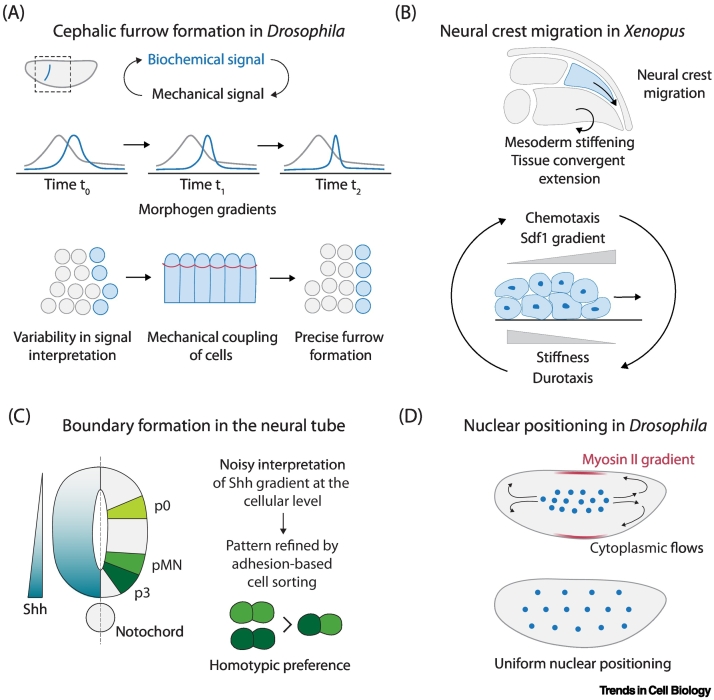
Robustness in development due to feedback. (A) Mechanical coupling of cells buffers against genetic noise to ensure the robustness of cephalic furrow formation with high precision in *Drosophila* [9]. (B) Feedback between chemotaxis and durotaxis regulates neural crest migration in *Xenopus* [13]. (C) Adhesion-based cell sorting underlies precise boundary formation despite noisy signalling in the neural tube in zebrafish [29]. (D) Myosin gradients and cytoplasmic flows position nuclei uniformly in the *Drosophila* blastoderm [41]. Abbreviation: Shh, sonic hedgehog.

Mechanics can couple cells and constrain their relative positions and behaviour, and therefore can improve patterning robustness by coordinating cell movements. Mechanical coupling of cells via actomyosin activity has been demonstrated to act as a denoising mechanism to accomplish precise tissue folding of the cephalic furrow in *Drosophila* gastrulation [9] (Figure 1A), conferring morphological robustness against transcriptional and mechanical noise. Tissue surface tension of somites maintained via integrin and fibronectin in zebrafish [10], and cellular rearrangements downstream of signaling-mediated patterning in *Drosophila* [11], ensure robust left–right symmetry, highlighting morphogenetic processes that can counter significant variability. Further, mechanical forces can provide active coupling for the morphogenesis of neighboring tissues via more active feedback involving mechanosensation. For instance, tissue flows involving the coordinated movement of cells and convergent extension in the head mesoderm of *Xenopus* embryos results in tissue stiffening, which triggers collective cell migration of the overlying neural crest cells [12]. As the neural crest cells migrate, they generate a stiffness gradient in the nearby placode tissue, which in turn guides the neural crest cell migration as they undergo durotaxis [13], illustrating how tissue-level mechanical feedback can couple morphogenetic movements (Figure 1B).

The physical and mechanical properties of tissues, including cell shape, size, and spatial organization, can influence patterning and how cells differentiate [14,15]. The surface area of cell-cell contacts in developing ascidians, which have stereotypic cell geometry across embryos, is a key determinant of cell fate specification [16]. Cell shape governed by growth anisotropy also progressively restricts fate specification in *Arabidopsis* patterning [17]. In addition, ensuring that cells can respond to mechanical changes in their environment can guide patterning to making sure that these processes are robust. Such feedback interactions involve integrating physical forces with molecular signaling and gene expression within cells and are evident in both animal and plant development [18,19]. For instance, in the developing incisors in rodents, cell proliferation generates forces that are resisted by the surrounding tissue, and the resulting compressive stress and mechanical anisotropy in the tissue activate intracellular Yap to specify the enamel knot signaling center [20]. In the mouse lung, **extracellular signal-regulated kinase (ERK)** (see Glossary) signaling activity in areas of high epithelial curvature promotes apical actin polymerization to reduce curvature, thus forming a negative feedback loop that regulates repetitive branching patterns [21]. During avian gastrulation, the contractile forces generated by a prepatterned posterior domain of the embryo enhance local expression of a secreted molecule, **growth differentiation factor 1 (GDF1)**, as well as create long-range tension that represses GDF1 at a distance [22]. This ensures self-organization, which is robust to perturbations such as embryo bisection. Further, mechanics can influence cell fate choices not only through direct force transduction, but also indirectly by modifying tissue geometry. In the mouse intestinal epithelium, the tissue curvature found in the villi *in vivo* concentrates diffusive morphogen signals to localize and restrict stem cell fate [23]. Further, externally imposed tissue curvature *in vitro* alone can bias the spatial location of symmetry- breaking, ensuring that intestinal stem cells reside in specific regions [24].

Overall, the contribution of mechanical feedback to ensure robustness can be generalized to provide both immediate physical responses to defects or perturbations and regulatory adjustments over longer timescales through gene expression changes, establishing safeguards against developmental variability and errors.

### Feedback from pattern formation to mechanical forces

Conversely, patterning can also change the mechanical forces in tissues to ensure that shape and form are robustly coordinated. In the developing inner ear epithelium, cell fate acquisition generates localized patterns of actomyosin tensions that can correct relative cell–cell positions [25,26]. Asymmetric divisions of blastomeres in the mouse embryo result in unequal inheritance of apical polarity, thus coupling cell position and fate as the apical domain reduces contractility and induces **trophectoderm (TE)** fate in outer cells [27,28]. Further, morphogen signaling, in addition to its classical role in cell fate patterning, can have a more direct effect on mechanical properties of cells to increase patterning robustness. In the neural tube, a **sonic hedgehog (Shh)** morphogen gradient regulates the expression of cell adhesion molecules, thus linking fate specification and patterning [29] (Figure 1C). During zebrafish gastrulation, a Nodal signaling gradient in the mesendoderm regulates both an active motility gradient and differential adhesion between leader and follower cells to preserve patterning during 3D collective migration [30].

### Feedback involving morphogenetic flows

Another emerging feedback is how morphogenetic flows can modify the spatial arrangement of cells, and accumulating evidence suggests that cellular movements can either disturb patterning or contribute to robust patterning refinement [31]. Although patterning is often studied in the context of static reaction–diffusion models, cells extensively move and rearrange during morphogenesis even after fate commitment [32]. Cell rearrangements via differential adhesion contributes to precise boundary formation during neural tube patterning [29,33] and specification of the midbrain–hindbrain boundary [34]. Likewise, cell sorting underlies patterning of the **inner cell mass (ICM)** in mouse blastocysts [35,36], anterior–posterior patterning of somites [37], and symmetry breaking in gastruloids [38,39]. During zebrafish axis extension, more global cellular flows favor patterning by changing the distance between opposing signaling poles [40]. Besides tissue flows, cytoplasmic flows can also result in the formation of high-order self-organized patterns (Figure 1D). For example, cytoplasmic flows in the *Drosophila* syncytial blastoderm driven by graded myosin contractility ensure uniform spatial distribution of nuclei, required for synchronization of the cell cycles during early development [41].

## Developmental robustness via optimal coupling of biological parameters

Robustness in tissue morphology and patterning may arise as a result of physical constraints on cells and tissues. For example, many tissues have a mesoscopic organization that is reminiscent of foams because this is the energy minimum of surface tensions, irrespective of the biological mechanisms [42,43]. This can allow the canalization of different biological regulatory mechanisms into a few stable morphologies, an idea that dates as far back as D’Arcy Thompson’s seminal work *On Growth and Form* a century ago [44].

Not all living systems may have feedback mechanisms and cell fate plasticity. During development, patterning robustness against natural variability in tissue size can be achieved through morphogen gradient scaling. The formation and scaling of morphogenetic gradients during early embryo development is one of the first processes to have been investigated quantitatively in terms of robustness [45,46]. The Bicoid gradient [47,48] scales with embryo length not only within *Drosophila melanogaster* [49,50] but also across dipteran species, in which embryo sizes vary up to fivefold [51,52]. While scaling of the Bicoid gradient within a species is achieved through altered *bcd* mRNA deposition in the anterior of the oocyte, and therefore volume-dependent Bicoid production [49,50], scaling across species is proposed to have a distinct mechanism. Evolutionary adjustment of the protein lifetime may underlie the scaling of the gradient across species, in addition to the spatial distribution of nuclei in the embryo [51,52]. Stem cells in a growing tissue may specify cell lineages with specific proportions before the tissue grows and transforms its shape. This would impose a challenge to the system, as tissue growth and morphogenesis are inevitably imprecise at the cellular scale and may not fit with the numbers and types of differentiated cells for precise patterning. How robustness and precision can be ensured in such systems lacking feedback and cell fate plasticity remains an open question. In this section, we highlight such cases and propose a new framework to understand developmental robustness.

### Robustness of cell fate specification

Robustness of qualitative traits such as cell fate patterns is challenging to evaluate, but it can be investigated by thoroughly quantifying phenotypic variation, as demonstrated by work on patterning robustness in *Caenorhabditis elegans* [53]. In adult *C. elegans* worms, the vulval cells form the copulatory organ in hermaphrodites and also have an egg-laying function. They are specified during the larval stage of development, from a stereotypic pattern of six epidermal cells, as a result of intercellular communication through the **epidermal growth factor (EGF)–Ras–mitogen-activated protein kinase (MAPK)** and **Notch signaling pathways** [54]. Combining controlled modulation of the EGF and Notch signaling pathways with an extensive analysis of the vulval cell fate pattern, a remarkably robust feature of these cells was identified [53]. The vulval cells can tolerate a fourfold variation in the genetic dose of the upstream signaling molecule EGF without altering the cell fate pattern, beyond which patterning errors become apparent. An invariant cell fate pattern with high precision was achieved within certain limits of *lin-3* and *lin-12* expression, while deviations from this limit caused patterning errors, suggesting that an ‘optimal’ window of gene expression is crucial for robust patterning. A more recent analysis of this process with EGF and Notch signaling as system parameters described a geometric model without coupling the two pathways, which could predict experimental cell fate outcomes at transition zones, in addition to defining which combinations of signaling strength permit or forbid cell fate transitions in the parameter space [55]. Furthermore, while a number of studies on the precision of cell fate domains have focused on prepatterned cues, current theoretical work has started to define utility functions that quantify the robustness of self-organized patterning [56]. Importantly, there is often a trade-off between achieving high reproducibility (making the same pattern reliably) and generating complex patterns, since more elaborate or detailed structures are typically harder to reproduce consistently. This reflects the need to optimize both pattern reproducibility across embryos and pattern complexity, and the utility function approach provides a quantitative informational metric with which different types of patterning mechanisms can be compared.

### Coordination between tissue growth and patterning

**Figure 2:**
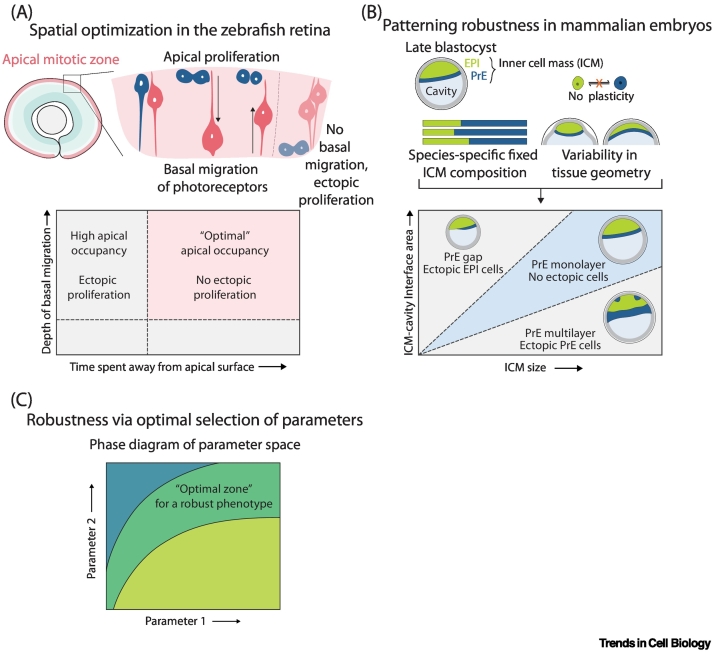
Robustness in development in the absence of feedback. (A) Spatial optimization in the developing retina. Basal migration of photoreceptor cells prevents cell crowding at the apical mitotic zone and maintains optimal cell occupancy to allow cell proliferation at the apical side [58]. (B) Inner cell mass (ICM) patterning in the mammalian blastocyst. The fixed proportion of cell types in the ICM is species specific and optimal for patterning precision in a tissue of particular size and geometry in the absence of cell fate plasticity [62]. Parameter space and ‘optimal’ zones. Illustration of a 2D phase diagram of a parameter space with an optimal zone defined by the values of the two parameters. Robust outcomes can be achieved through optimal selection of coupled parameters. Abbreviations: EPI, epiblast; PrE, primitive endoderm.

Robust development requires the consistent formation of organs with reproducible sizes. This consistency can emerge from heterogeneous cellular processes, where individual variability is buffered through statistical averaging. Spatiotemporal averaging of variability in cell growth rates results in robust sepal size in plants [57]. The coordination between tissue growth and pattern formation is critical, as patterns need to be established and maintained through ever-changing conditions as embryos grow. For example, in the developing vertebrate retina, neurons are organized into distinct layers as they form, creating a multilayered tissue architecture essential for function. This patterning occurs simultaneously with cell proliferation, necessitating the maintenance of the overall structure as the tissue grows. Photoreceptor migration in the zebrafish retina was reported to play an important role in coordinating tissue growth and cell lamination [58] (Figure 2A). During the peak of neural progenitor cell proliferation, photoreceptors migrate basally and remain there before migrating back to the apical side. This basal photoreceptor migration is required for proper cell divisions at the apical side. Quantification of migration trajectories and the duration and basal depth of photoreceptor migration has revealed key system parameters that are crucial to allow cell proliferation at the correct position in the tissue. Photoreceptor cells must migrate away from the apical surface for a minimum duration to ensure proper cell occupancy at the apical mitotic zone. Once this duration is met, the exact distance they travel basally beyond a minimum threshold becomes less critical for maintaining optimal apical occupancy. It is likely that, in the absence of tissue-level feedback, the time cells spend away from the apical side could have been under evolutionary constraints. This exemplifies how the duration of basal migration can allow effective ‘crowd control’ to prevent congestion and optimize apical occupancy of photoreceptor cells and ultimately allow proper patterning of the retina with tissue growth.

### Patterning robustness during embryo development

Early mammalian development is a well-studied example of robust cell fate specification and patterning through variabilities. The ICM of the developing mouse blastocyst is patterned such that the **primitive endoderm (PrE)** cells form an epithelial monolayer separating the pluripotent epiblast (EPI) from the blastocyst fluid cavity. The transcription factors **NANOG** and **GATA6** form the gene regulatory network that specifies EPI and PrE cell fates [59., 60., 61.]. While initially heterogeneous gene expression allows cell fate plasticity, it is gradually lost as the precursors become committed to a particular fate by the late blastocyst stage. Quantitative analysis of EPI and PrE cell numbers in embryos of varying sizes has revealed that the ICM has a fixed proportion of EPI/PrE cells with an apparent lack of cell fate plasticity in late blastocysts [62] (Figure 2B). The fixed proportion of cell types is consistent across embryos with varying sizes as cell numbers scale linearly, whereas the tissue surface:volume ratio scales nonlinearly, thus challenging the precision of pattern formation with the occurrence of ectopic cells in larger or smaller embryos. The three key observations – fixed cell-type proportions, naturally variable tissue size and geometry, and absence of cell fate plasticity – form the basis for an alternative mechanism to achieve patterning robustness. Comparative analysis of mouse, monkey, and human blastocysts [62], whose fate proportion, ICM size, and geometry differ, led to the concept that coupling these parameter values can ensure patterning robustness and may be conserved at the evolutionary timescale. An upstream signal that controls both ICM fate proportion and geometry may couple these parameters. For instance, modulating FGF signaling in the mouse ICM not only changes the cell fate proportion in the ICM [61,63,64], but also cell shape, physical properties and tissue geometry [65], making such signaling a potential candidate [64., 65., 66.].

### Emerging concept of optimality

A common theme emerges across these studies: fine-tuning and coupling of key underlying parameters through evolutionary adaptation can confer robustness. In these cases, when one parameter varies within or among species, others can compensate, thus maintaining functionality. Embryos may occupy a ‘sweet spot’ in *n*-dimensional parameter space, where *n* is the number of parameters underlying morphogenesis and patterning (Figure 2C). The vulval cell fate map as a function of signaling factors *lin-3* and *lin-12* [53] and the ICM–cavity interface area as a function of EPI/PrE cell number and proportion [62] exemplify such parameter spaces. Optimal selection of coupled parameters such as cell fate, proliferation, and mechanics can thus be a means to achieve patterning robustness in tissues that inherently possess variabilities but have limited plasticity. To study the potential role of such an optimality in selecting parameter values, however, it needs to be first investigated and defined regarding which functional outputs (e.g., patterning precision, growth, and/or intake of nutrition) should be optimized in specific biological contexts of interest. Optimization principles may be responsible for robust outcomes not only in tissue patterning but also in other developmental processes. The concept of optimality has been studied in multiple fields (Box 1), and with quantitative analyzes it could be applied to more developmental contexts.

Box 1 **Optimality in theory**

Optimality theory has been extensively applied to ecology and evolution [77,78], weighing the costs and benefits of specific features to test whether or not a behaviour is optimal. Compared with disciplines like economics, one key outstanding issue is to define the variables to be optimized, and determine how to measure them and to weigh costs and benefits that can be of very different nature. Recently, this concept has been extended to biology. For instance, in the context of genetic networks [79], optimality refers to the amount of information that propagates from specific morphogen signals towards patterns of cellular fate via decoding. In the context of bioenergetics [80,81], a number of phenomenological laws, including Kleiber’s law, have been observed across a number of species but also measured over development [80., 81., 82., 83.], and have been proposed theoretically to derive from metabolic optimization [84]. More generically, a key question is whether and how organisms optimize multiple tasks and functions at the same time. The concept of Pareto optimality has been put forward to describe this, where many different phenotypes across species are plotted in a specific morphospace [85], parametrized from first principles or from dimensionality reduction methods, to see whether they occupy well-defined regions. For instance, if two traits are optimized for with different weights, all phenotypes should arrange in a 1D line linking two extrema, where only one of the traits is optimized. How to generalize these ideas to developmental biology, where high-dimensional morphogenetic trajectories are observed, remains an open question, which may provide more insights into the underlying mechanisms of embryo patterning and morphogenesis.

**Optimization of energy allocation**

Embryos have a fixed pool of energy and resources, or ‘energy budget’, and need to allocate resources to various processes during embryogenesis. The energy budget of embryogenesis can be quantitatively measured, with *Drosophila* embryogenesis using approximately 10 mJ of energy [86]. How do embryos allocate these resources and optimize processes? What other developmental constraints are embryos under? Recent work has explored the energetic costs of developmental processes [87,88]. For instance, *Drosophila* embryos start out with a limited amount of energetic resources, and this limit is crucial for the deceleration of nuclear division cycles to facilitate zygotic genome activation and the progression of gastrulation [89], emphasizing the importance of optimal resource allocation to ensure developmental progression.

Alt-text: Box 1

**Figure 3:**
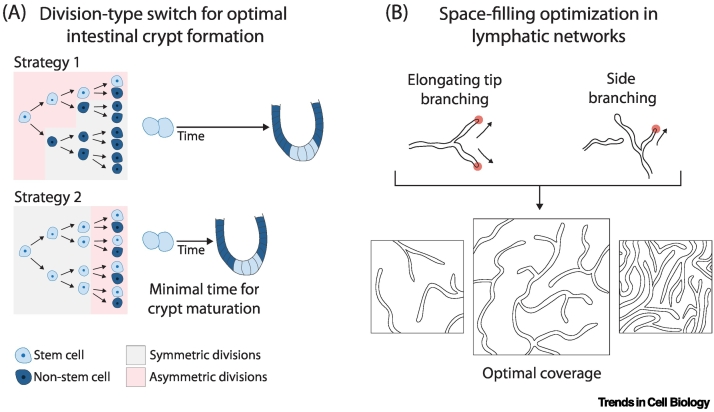
Optimization in various systems. (A) Optimization of time in intestinal crypt formation. Stem cells first undergo symmetric divisions to expand the stem cell pool, which is followed by a switch to asymmetric divisions (Strategy 2) to increase the differentiated cell population. This minimizes the time duration to form a mature crypt [69]. A mature crypt typically has four to six stem cells that generate six differentiated epithelial cell types [70]. (B) Optimal coverage in lymphatic branching networks can be achieved through side branching in combination with branching of elongating tips [76].

Geometrical parameters of cells including size, shape, and division dynamics could be highly amenable to optimization, as cell shape changes are fundamental to morphogenesis. In eight-cell mouse embryos, reducing the variability of cell division timing disrupts morphogenesis and ICM-TE patterning, suggesting that cleavage asynchrony facilitates energy minimization of embryo morphology by packing [67]. Given that high variability in division timing would be incompatible with the limited duration of the eight- and 16-cell stages, it is conceivable that this degree of variability is optimal for the generation of a sufficient number of ICM cells by packing embryos within the defined time frame. During symmetry breaking of the intestinal mesenchyme prior to villus formation, the distance between cell clusters that will initiate the site of villus formation may be optimal to maximize the number of villi formed thereafter [68]. Optimization could also be evident in terms of developmental timing, as embryogenesis requires certain processes to be completed within a particular time frame to allow the timely progression of subsequent steps. For example, in the intestinal epithelium, the time required to make a fully mature crypt is minimized by generating the necessary cell types through ‘bang-bang’ control (Figure 3A) [69]. Intestinal crypts prioritize the establishment of the complete stem cell pool first through symmetric divisions, then switch to asymmetric divisions to expand the differentiated cell population and promote tissue growth. This strategy minimizes the time required to establish a mature crypt, which typically contains four to six stem cells capable of generating six distinct differentiated epithelial cell types [70].

Finally, optimization of tissue shapes has been studied extensively in the context of branching networks, where a key output can be the maximal rate of fluid transport, or minimal energy expenditure associated with transport through the network. In several glandular organs, including the mammary gland, salivary glands, or pancreas, as well as in *Drosophila* sensory neurons, reconstruction of the entire branch network has revealed branching patterns according to a simple set of rules [71., 72., 73., 74., 75.]. Interestingly, additional rules based on local sensing and/or mechanical interactions have been proposed to allow space filling optimization. Lymphatic networks are optimized during late development by local side branching, which targets low-density regions to rapidly reach tilings with low spatial fluctuations [76] (Figure 3B).

## Concluding remarks

To investigate the potential role of optimal selection of coupled parameters in robustness, biological systems, functional outputs, and experimental approaches must be defined. First, developmental processes *in vivo* need to be quantitatively characterized so that robust outcomes of complex, dynamic processes can be documented. This requires high-throughput measurement of both spatial and temporal dynamics of underlying parameters at the cellular and tissue scales, such as the direct measurement of cell physical parameters or their estimation using quantitative image analysis. This must also be done with high statistical power and in controlled genetic and environmental conditions, to understand the different sources of variability in the system. From a theoretical perspective, this also means going beyond modelling morphogenetic processes as noise-free minimization of energies – as often done in vertex models, for instance – and considering systematically different sources of stochastic noise and cellular heterogeneities. These different possible sources of variability must then be inferred from data. Computational modelling integrating experimental data is then extremely useful, not only to simulate developmental processes under various parameters and noise levels but also to predict how changes in one parameter affect others and the overall system stability. Second, it is key to define systematically robustness and optimality; for instance, by clearly specifying the quantitative trait that is assessed or the types of noise and variability that need to be buffered. Third, to test experimentally whether robustness arises from feedback or by optimal selection of coupled parameters, dissection of the response of the system to controlled perturbations is necessary. This can involve precise variations in gene expression as opposed to simple knockouts or overexpression, or modulation of cellular pathways with optogenetic tools. Importantly, perturbations over a certain range of the parameters are necessary to determine an optimal zone for the phenotype or response of the system by constructing a multiparametric perturbation landscape, or ‘morphospace’. For example, in zebrafish retinal patterning, modulating the duration of photoreceptor basal migration and analyzing the number of cell divisions in ectopic locations in response would help to confirm whether a certain interval of time is optimal for tissue growth and morphogenesis [58]. The onset of the peak of cell proliferation and duration of photoreceptor migration may also have been fine-tuned through evolutionary adaptation.

Overall, the field of developmental biology is currently at a pivotal juncture, where rapid innovations in live imaging with high spatiotemporal resolution, machine learning for multiscale segmentation, and biophysical modelling give us an unprecedented view into the development of living organisms, with which we may discover more mechanisms underlying the robustness of living systems (see Outstanding questions).

Outstanding questions To investigate the mechanisms underlying robustness and precision in development, experimental systems and functional outputs must be defined in the specific process of interest. Which developmental process is to be addressed, with which functional outcome, involving which variabilities at which spatiotemporal scale?

Developmental processes *in vivo* need to be quantitatively characterized so that robust outcomes can be documented. This must be done with high sample number and statistical power, and in controlled genetic and environmental conditions, to understand the different sources of variability in the system. Which parameters are relevant to the process and outcome of interest? How can they be measured in a large quantity and in a (semi)automatic manner?

Theory needs to go beyond the modelling of morphogenetic processes as noise free, and consider systematically different sources of stochastic noise and cellular heterogeneities. What types of models need to be built, including noise? Which noise should be considered? How can different noise types be inferred from experimental data?

The response of the system to controlled perturbations needs to be dissected to find whether robustness arises from feedback or by optimal selection of coupled parameters. Perturbations over a certain range of the parameters are necessary to determine an optimal zone for the phenotype or construct a multiparametric perturbation landscape. How can each parameter be modulated, ideally with spatiotemporal control and without influencing other parameters?

Alt-text: Outstanding questions

## Glossary

**EGF–Ras–MAPK signalling pathway**: a signalling cascade initiated by the binding of EGF to its receptor (EGF-R), leading to the activation of Ras, a small GTPase. This activation triggers a series of phosphorylation events involving the MAPK pathway, resulting in cellular responses such as proliferation, differentiation, and survival.
**Extracellular signal-regulated kinase (ERK)**: a key protein in the MAPK signalling pathway that regulates cellular processes such as growth, differentiation, and survival.
**GATA6**: a transcription factor involved in the regulation of cell differentiation and during development.
**Growth differentiation factor 1 (GDF1)**: a protein belonging to the transforming growth factor beta superfamily, involved in mesoderm induction during embryonic development.
**Inner cell mass (ICM)**: a cluster of cells inside the mammalian blastocyst that gives rise to the embryo proper and differentiates into the epiblast and PrE.
**Mitogen-activated protein kinase (MAPK)**: a type of serine/threonine-specific protein kinase involved in directing cellular processes.
**NANOG**: a transcription factor responsible for maintaining pluripotency and self-renewal of embryonic stem cells.
**Notch signalling pathway**: a highly conserved cell–cell communication pathway that regulates cell fate decisions during development and tissue homeostasis, involving interactions between the Notch receptor and its ligands, such as Delta.
**Primitive endoderm (PrE)**: epithelial cells in the mammalian blastocyst that form extraembryonic tissues such as the yolk sac.
**Ras**: a family of small GTPase proteins involved in transmitting signals within cells to control cell growth, differentiation, and survival.
**Sonic hedgehog (Shh)**: a signalling molecule that regulates organ formation during embryonic development.
**Trophectoderm (TE)**: outer layer of epithelial cells in the mammalian blastocyst that forms the placenta.
